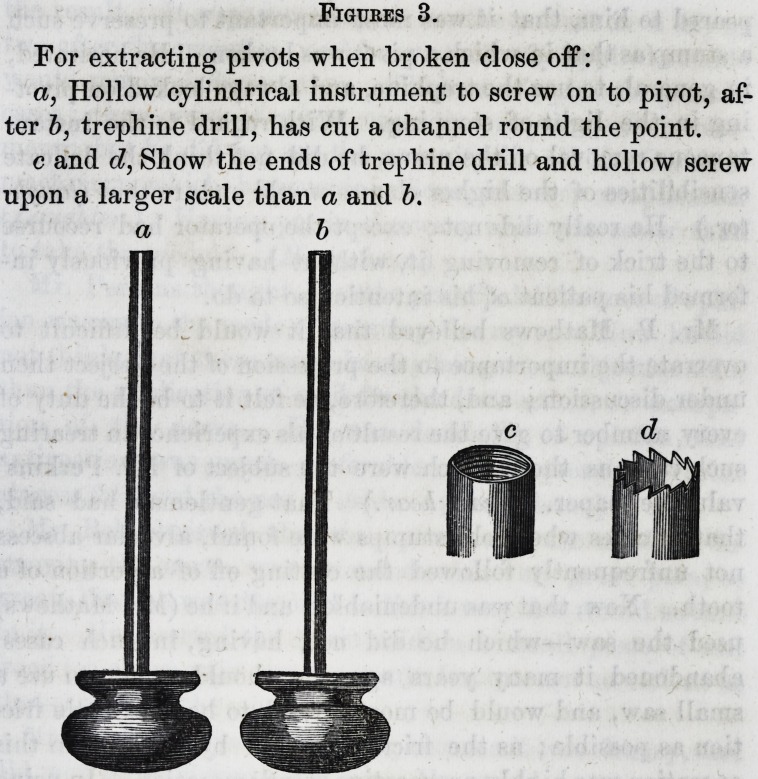# College of Dentists.—Special Meeting, July 30, 1857

**Published:** 1858-01

**Authors:** 


### ARTICLE XV.

### *College of Dentists.—Special Meeting, July* 30, 1857.

A SPECIAL meeting of the members of the college was held on Thursday, July 30th, 1857.

*Election of Members.*—The following gentlemen were balloted for and duly elected members of the college: P. J. Boulger, Esq., Norwich; D. G. Clerk, Esq., Calcutta, India; W. S. Clerk, Esq., Calcutta, India; —— Feltham, Esq., Jersey; —— Grey, Esq., Sheffield; G. F. Hare, Esq., Limerick; G. F. Harrington, Esq., Isle of Wight; E. King, Esq., Brecon; F. Lloyd, Esq., Agra, India; J. E. Rose, Esq., Liverpool; J. F. Rose, Esq., Preston; W. D. Woodburn, Esq., Glasgow; W. S. Woodburn, Esq., Glasgow. Several other candidates were proposed and seconded.

During the evening, Dr. R. Reid, one of the Vice-Presidents, read two papers—one upon the "*Alveolar Hemorrhage Compress*," and the other "*Observations on the Dentition of the Lilliputian Aztecs*." Our space will not permit your giving the papers in the present number. They shall, however, receive due attention in our next.

We must also mention, that Dr. Reid very generously presented to the Museum of the College a model with the compress attached: and also various casts illustrating the dentition of the Aztecs.

Those papers were received with much applause. The President thanked Dr. Reid for, not only his excellent papers, but also the unexpected pleasure they had in having him present on this special occasion. The company then proceeded to the Museum, where they partook of tea and coffee, &c.

*Remarks on Pivoting Teeth, &c.*—The following is the paper read on this subject by Mr. Perkins, at the monthly meeting held on the 5th May:

The operation of pivoting teeth, although pretty well known and practiced, does not appear to have received all the consideration which its importance and consequences seem to entitle it to.

As I intend to devote a little time to the subject, it may be as well to describe the operation first.

To begin at the beginning, I think, is always the best way; for, however well a subject may be known, or be supposed to be known, there generally happens to be some persons who do not quite understand *all about* it; and when a thing is considered ordinary or common, many do not like to make any inquiry on the subject for fear of being considered ignorant, and by that means, remain in the dark rather than ask for information. I have, myself, not unfrequently, been much disappointed in referring to very well written works for information on some so-called common subject, by finding it asserted by the author that the subject "is so well known" that there is no need of describing it. Now, as a description of this common and well-known thing was what I sought and failed to find, and feeling that I, in all probability, was not the only person who needed information in the like case, I could not help wishing, that the author had taken more pains to fully describe all subjects he touched upon; as in that case, the ignorant would have been instructed, though the wise would not have been compelled to read; or, if they *had read*, might have found something satisfactory to them, either by confirming their previous view, or by setting them to think upon the different, and to them, new view taken by the author. It is, in short, far better to say nothing at all on a subject, than so little, as to leave it unintelligible.

The operation of pivoting becomes necessary when the crown of a front tooth is so far decayed as to be useless, or decidedly unsightly. The first thing to be done, is to cut off the remaining part of the crown, which (if not of too substantial a character,) may be accomplished by the cutting or excising forceps; but when the part to be removed is considerable and solid, a saw, or thin half-round file must be used in such a manner as to avoid touching the centre of the tooth, cutting first one side, and then the other, so as to leave little more than the centre untouched, by which means, a very great deal of unnecessary pain will be avoided. The crown may then be detached by means of the excising forceps, and a small soft broach, prepared for the purpose, should be ready, and quickly and steadily passed up the opening in the tooth, when, with a few turns of the instrument, the vessels will be separated, and sensibility in the part cease. After having filed the stump level with the gum, arching or hollowing it so as in a measure to follow the line of the enamel of the removed crown, the cavity may be broached to the proper size for receiving the pivot, the impression taken, and the case completed.

I would here observe that, in my opinion, it is never advisable to cut off a tooth and pivot a new one to the stump at one sitting, as in case of inflammation taking place, which is not at all unfrequent, even in favorable cases, the tightness of the pivot, not only adds to the inflammation, and prevents the discharge of pus (should any be secreted) but causes a very great deal of pain, and bids fair to render all your previous work useless, by establishing an abscess and so necessitating the removal of the stump; whereas, had the fixing of the new tooth been deferred for a day or two, or until the swelling and sensitiveness had subsided, the new tooth might have been fixed with the most perfect satisfaction to both parties concerned.

I am induced to make these observations, from knowing that there exists some difference of opinion on this subject among dentists; some holding the opinion which I have here expressed, while others certainly *act* upon a different theory, whatever their opinion as to the propriety of such a course may be. I may just mention here, that it sometimes happens, that after the crown of a tooth is removed, the nerve remains still unexposed, owing to the crown not having been cut off sufficiently high up, or to an unusual amount of solidity of structure in the tooth, whereby the internal canal is reduced, both in length and diameter; at the same time, the centre is so exquisitely sensitive that it becomes a matter of difficulty, if not of impossibility, to proceed, on account of the pain caused to the patient by the filing that is necessary to complete an opening for the instrument to be used in the destruction of the nerve; the difficulty, however, which here presents itself, may be readily overcome by the careful application of a small quantity of nitric acid.

The method I make use of is as follows:—I carefully dry the surface of the stump with cotton, wool, or lint, and then as carefully apply a camel's-hair pencil, dipped in nitric acid, to the spot indicating the entrance to the canal. A little instantaneous pain is all that is usually felt, and in less than a minute I am enabled to proceed with the filing until an opening sufficient for my purpose is effected; and should the stump become sensitive again, before the object has been attained, a second or third application may be made without any inconvenience to the patient, or chance of injury to the neighboring teeth, provided the part is carefully dried previous to applying the acid : the drying being absolutely necessary, as in the first place, it prevents the acid going over a greater surface than it is intended to affect, and secondly, by its being undiluted, the effect which you wish to produce is done more speedily and effectually.

I have before stated it to be my opinion, that in describing anything that is worth describing, it is better to omit nothing that can be useful or necessary, under the impression that it is too well known to need a description, and in accordance with that opinion, I will venture a few remarks upon the method of taking an impression for a pivot tooth; as I believe all operators will agree with me, that the best work, made to an incorrect model, is time, ingenuity, and labor thrown away, producing nought but vexation and disappointment, both to patient and operator. Now, the method that I have seen most frequently used, and indeed the one that I myself have many times used, is this : after having bored the stump, a small piece of boxwood, of rather less than a quarter of an inch in diameter, and from three-quarters to an inch long, is shouldered down at about a quarter of an inch from one end, and reduced to the size of the hole bored in the stump; it is then placed in a small quantity of softened wax, with the pin projecting through the upper surface ; the pin is then entered into the pivot hole, and the wax pressed carefully up, so as to get the necessary impression ; and if drawn away without shifting or altering the position of the peg or pin, this method answers very well ; but it is in drawing away the impression from the mouth *without altering the position of the pin,* that all the difficulty lies. I have often succeeded very well with the above method ; but I have also had a great deal of extra trouble at times, owing to the unperceived alteration of the pin, in withdrawing the impression from the mouth. I, therefore, with a view to save myself trouble, and to render the operation more certain, have contrived a method which is certainly a great improvement over that one I have described, and if not quite perfect, is, I think, as perfect as is necessary for the purpose, in the hands of any moderately skillful and careful operator.

I will endeavor to describe it.

I have a short tube, say a quarter of an inch long, its external diameter about the eighth of an inch, its internal diameter the size of wire used for pivoting; the outer part is screwed, and to one end is soldered a small round or oval plate, the screwed part is furnished with a nut. I have then a small tray, say large enough to cover six teeth, its depth not more than a quarter of an inch; at the distance of a quarter of an inch from the inside of the external circle, I have three or four holes pierced, into either of which I can fasten my small tube by means of the nut. I have a pin of silver wire fitting accurately, but not too tightly, into the tube, and also fitting easily into the hole in the stump to be pivoted.

The method of application is simple. The tube being made fast into the hole in the tray which will best answer your purpose, you fill your tray with wax in the usual way, and pass your pin through the tube from the underside, sufficiently beyond the wax on the upper side, to enter the hole intended for the pivot; you then apply it to the mouth. Entering the pin clearly into the hole in the stump, and pressing up the wax carefully, in a plane perpendicular to the direction of the pin, you get a very accurate impression; the pin may be thoroughly pushed home at the same time. The impression thus taken may now be withdrawn with scarcely a possibility of deviation, even with a careless modeller, the tube effectually preventing any deviation taking place in the position of the pin, while the tray insures the accuracy of the impression in other respects.

As it occasionally happens, that previous to fitting in a new pivot tooth, the old pivot remaining fast in the stump has to be removed, and as, if it should happen to have been broken off close to the gum, it is not always so simple an affair as at first sight it would appear to be, it would not I think be out of place here, if I were to describe the method of getting over this trouble, in what I conceive to be the shortest way possible.

There are I believe a great many ways of surmounting this difficulty, all more or less ingenious, according to the amount of difficulty to be overcome, or the power of invention developed in the artist by the necessity in which he is placed. I will not, however, trespass on your time to describe the methods which are generally known, but the one which I have found and believe to be the best and ready method.

First, you need a hollow drill, a small trephine in fact, the hollow rather less in diameter than the pivot to be extracted. This instrument is, or was sold, years ago, by Messrs. Ash, and I dare say at other dental depots. But another instrument is wanted to finish the work, which the trephine has begun; and this instrument I have never seen anywhere except at my own house, and I believe is not generally known, and that is why I venture to describe it.

It is also a hollow cylinder; its outer diameter the same, or rather less than that of the trephine drill; the hollow in its centre is tapped, so that it forms a screw cutter, following readily on to the pivot, which the trephine has exposed; it must be hardened and tempered. You have then only to use it carefully with a little moisture, as if you were screwing an ordinary piece of wire, until you have got two or three turns of the screw on to the fast pivot, and you are enabled to extract it beautifully, with the greatest ease.

I have hitherto spoken of pivoting only in its simple and favorable form, or under favorable circumstances; but circumstances occur under which, however *desirable* the operation may be, success is almost impossible; sometimes arising from constitutional or physical causes, sometimes from inattention to the thorough destruction of the nerve, or separation of the vessels in the pulp cavity, and sometimes from the form of the root itself.

The case which I deem most favorable for the operation, may be described as follows: first, nerve unexposed, alive and healthy; and secondly, the fang straight with plane or horizontal section of a rounded equilateral triangular form, the pulp cavity being about central or equidistant from every point in the circumference; the chief thing to be observed is to send the broach, or instrument, thoroughly home so as to entirely separate the vessels inside the tooth from those connected with the periosteum, &c. If this be not completely done so as to extirpate the vessels from the inner cavity, although you may be able to complete your operation, you will find in most cases that the lacerated vessels will suppurate, and communicate their disease to the periosteum, and you will either have an abscess in the alveolus, that will cause the part to swell with great pain, or it will discharge itself between the socket and the root into the mouth; the latter is the least painful effect of the two, but in either case nothing but the extraction of the root will effect a cure.

The second case I shall allude to, is where the nerve is exposed and aching, the root well shaped and otherwise favorable. In this case there is little more to be observed than in the first case, viz. the thorough extirpation of the nerve from the pulp cavity, and rest for a day or two, that the irritation caused by preparing the stump may subside, previous to fixing the artificial tooth.

The third case that occurs to me is when the crown is gone, with more or less decay of the stump and softening of the bone, with no pain and with no *perceptible* discharge; generally, this kind of stump can be drilled without causing any inconvenience whatever; but this symptom, though flattering to the patient, is by no means a guarantee of success, for very frequently inflammation takes place with swelling and a good deal of uncomfortableness, if not actual pain. This will however, often subside by itself. I have, nevertheless, occasionally found a leech applied to the part of great service in reducing the inflammation, while the application of a little tincture of opium by means of a camel's-hair pencil to the part afterwards, is exceedingly useful and beneficial in subduing the pain, and helping to bring about a cure; but it sometimes happens that pus is deposited and the extraction of the stump becomes necessary; or, avoiding the extraction, you are compelled to make use of a plate instead of the neater way of pivoting.

The next case is where all the characteristics of the preceding case appear, and in addition a decided discharge of pus. If anything is to be done here, it is extraction; pivoting is out of the question.

I now come to a class of teeth that is occasionally met with, possessing all the characteristics of the first case named; differing only in the peculiar formation of the fang, the horizontal section of which I would describe as a compressed irregular oval. This form is met with more frequently I think in the superior central incisors than any other teeth. The great difficulty in dealing with this kind of formation, is the width and thinness of the pulp cavity, which resembles as much as possible the tube in a thermometer, without its regularity of form, rendering it exceedingly difficult, if not impossible, to pass any instrument, however thin, sufficiently far up the cavity, to separate the internal from the external vessels of the tooth, and which as I have stated before, I believe to be absolutely necessary for a perfect operation. I recollect some years ago, cutting off two superior central incisors for a lady, giving her comparatively little pain during the operation. The nerve in each was destroyed, and she went away perfectly comfortable; but owing to the thinness of the canal, I could not pass the broach, although a thin one, sufficiently far up to satisfy me; however, she only felt the smart shock from each as is usual in such cases, and all seemed to be over. But in the course of two days after, she returned to me saying that she had been suffering dreadfully; I advised leeching, which was complied with, but to little or no purpose, as they both suppurated, the pulp coming down to the orifice in the stumps, while pus was discharging from the alveoli of both, and I was obliged to extract them. They were of the kind to which I have here alluded; in addition to which, they were crooked or wavelike, adding as a matter of course to the difficulty of the flat cavity. I felt convinced at the time that this peculiarity of formation was the cause of all the trouble. In this case, however, extraction was unavoidable, and the only disagreeable consequences resulting from it, were the pain for the time it lasted, and having to wait a few weeks instead of a few days for her teeth. I have since had some cases similar to the one I have here related, and with few exceptions have felt justified in attributing the failure to this peculiarity of formation.

It will have been observed that in the course of these remarks, I have more than once laid great stress upon the necessity of the thorough extirpation of the vessels from the internal canal of the tooth. My reasons for so doing are these: first, I conceive that if the vessels are completely drawn from the pulp cavity, the upper part from which they are separated will immediately retract, and close above the extreme point of the fang of the tooth; the vessels in retracting will also close the orifice that has been opened, by the rupturing or tearing of the vessel apart; by which means the passage of blood through it is at once stopped, and the chance of suppuration becomes extremely remote, if not absolutely impossible; at the same time the vessels of the periosteum of the tooth are entire, and the circulation is continued as heretofore; so that although sensitiveness and life are destroyed in the interior of the organ, the vessels covering the outside, and in connection with the alveolus, are still active; exactly as we sometimes observe in trees and shrubs of different kinds that are decayed or pithless in the centre, while the bark, or periligneum, if in this case I may be allowed the term, retains so much of its former vitality as to produce leaves and new shoots; and although we can understand perfectly well, that the tree, or the tooth, as the case may be, is not as perfect as it formerly was, we know at the same time that it is very different from a dead organ; and I believe that this is one great reason why some pivoted teeth last so many years to what others do. Again, supposing the vessels to be lacerated and driven into the upper and narrow part of the cavity, the natural consequence must be inflammation commencing in the cavity, and extending through the foramen of the fang by means of the wounded vessels, and consequently to the vessels of the periosteum; and if the inflammation so set up does not subside, either naturally or otherwise, suppuration takes place, and you have at once a case of alveolar abscess.

Some months since I read an account of an invention by Dr. Coghlan, of Wexford, for obviating the inconvenience arising from a secretion of pus in the pivoted stump. It consists in making use of a tube for the pivot, instead of a solid piece of wire, by which means the secreted pus is allowed to escape into the mouth, and the force-pump action which he conceives to take place in fixing a tight-fitting cylindrical pivot into a stump, with its conseqences, are avoided. He says that he has found it answer, and would confidently express his conviction, that the use of the tube will remove the odium from the operation of pivoting which has hitherto been attached to it.

I find that Dr. Harris, of the United States, mentions the same kind of invention by Dr. Elliot, who after having invented it, found that it had already been used in France, so difficult is it to conceive an original, or at least a singular and original idea on any known subject.

The use of a tube in the case of a secreting stump, is, in my opinion, a decided improvement; indeed, I think there can be no doubt about it, nor do I see any *objection* to it in *any* case; at the same time I must confess that I do not approve of pivoting to stumps from which there is any discharge, they are, to say the least of them, disagreeable and unhealthy, and I would not recommend them being made use of in any case. They ought to be removed; at the same time there are circumstances which occasionally occur under which we are obliged to retain and use these unwholsome things; and in such cases the tube is certainly a great advantage. Similar results may be produced by filing the sides of the pivot flat, and tapering it in a small degree, or by making a small groove or channel at the side; but the tube is, after all, the best and most complete method of forming an outlet from the cavity.

Wood is occasionally used for pivots instead of gold, and I believe more particularly by our American brethren; for my own part I cannot see any advantage in its use for this purpose. In the first place it is not so strong as gold; and secondly, it scarcely ever happens that the hole in the artificial tooth to be inserted, and the hole in the stump are in a straight line together; and as wood cannot be readily bent I think it a bad plan to use wood, except in old cases where the hole in the stump has become so large and taper as to require filing previous to the insertion of a metallic pivot.

In conclusion, I will just state that I have here recorded my own experience, with an opinion founded on that experience; others doubtless have had similar experience, and have formed similar or different opinions, according to the views they have taken, and the ideas suggested therefrom. Difference of opinion is the parent of argument. and argument if conducted with good sense and consideration on all sides, must ultimately produce the truth, one of the greatest and best objects of our existence; without which all our efforts to attain perfection will be but lost labor and misspent time.

FIGURES 1.

The tooth marked *a*, represents a tooth with the pulp *partly* extracted, the remaining part being lacerated, and forced into the upper and narrow part of the cavity, renders inflammation almost certain, and periosteal suppuration probable.

The tooth *b*, represents a tooth with the pulp *entirely* extracted from the internal canal, and the ruptured vessel closed and retiring above the fangs. In this case, inflammation is scarcely possible, there being no wounded vessels to affect the periosteum.

*c,* Horizontal section of unfavorable tooth for pivoting.

*d,* Horizontal section of ordinary or favorable tooth.

FIGURES 2.

For taking impression for a pivot tooth:

*a,* Hollow cylinder, with top plate and nut to fasten in wax tray.

*b,* Pin to pass through cylinder into the stump to he pivoted.

*c,* Plan of wax tray, drawn without the rim, which should not be more than a quarter of an inch deep.

FIGURES 3.

For extracting pivots when broken close off:

*a*, Hollow cylindrical instrument to screw on to pivot, after *b*, trephine drill, has cut a channel round the point.

*c* and *d*, Show the ends of trephine drill and hollow screw upon a larger scale than *a* and *b*.

A member observed, that he had found a great advantage to arise from the use of a solution of gutta percha, in preventing the saliva from interfering during the process of pivoting.

Mr. Rogers never hesitated in performing the operation in the case of a first bicuspid, and he had recourse to it when he met with a sufficiently healthy first molar. With regard to the nerve, he had always found that pivoting might be accomplished immediately after the total destruction of the nerve. He had never fixed a tooth, immediately after this destruction, that gave any trouble afterwards. They might have the instrument ready; for in taking a model, he had never found any occasion for the use of such apparatus as that described by Mr. Perkins. At the same time, it appeared to him, that it was most important to preserve such a stump as that in which a pivot was broken. He hesitated, in general, to use the trephine, and always looked on pivoting in the light of stopping. With regard to the instantaneous removal of the nerve, he did not think the delicate sensibilities of the higher classes would endure that (*laughter.*) He really did not; except the operator had recourse to the trick of removing it, without having previously informed his patient of his intention so to do.

Mr. P. Mathews believed that it would be difficult to overrate the importance to the profession of the subject then under discussion; and, therefore, he felt it to be the duty of every member to give the result of his experience in treating such cases as those which were the subject of Mr. Perkins' valuable paper. (*Hear, hear.*) That gentleman had said, that in cases where old stumps were found, alveolar abscess not unfrequently followed the cutting off of a portion of a tooth. Now, that was undeniable; and if he (Mr. Mathews) used the saw—which he did not, having, in such cases, abandoned it many years ago—he should prefer to use a small saw, and would be most careful to have as little friction as possible; as the friction caused by the saw in this operation was highly provocative of inflammation. In using the broach when dealing with the stumps of old teeth, great care was also requisite. He generally used a No. 4 or No. 5 square drill, with very sharp edges, and proceeded very slowly. But his mode of cutting the tooth was by an excising forceps, which fitted the crown of the tooth, and cut to the shape wanted, so that he never had any occasion to use the file, but after cutting, at once proceeded to apply the broach. (*Hear, hear.*)

Mr. Purland used a diagonal broach, and bored about three-fifths the length of the stump. He destroyed the nerve with nitric acid, applied on a piece of gold wire; and knowing the exact length of the stump, he made his pivot rather shorter, because the nerve dropped on the pivot, and where the latter was too long, inflammation was sometimes the result. It was necessary to see the patient in a day or two after the operation, and hot water freely applied outside would remove inflammation. That was his plan for general cases; there were, of course, cases that required other treatment; but he did not see the necessity for all Mr. Perkins' machinery, which, however ingenious, was very complicated. (*Laughter.*) Having put in the wax, what was easier than to take the model? (*Hear, hear.*)

Mr. Perkins thought, that the principal difference of opinion was as to the mode of treating the nerve. Now he did not think, that there could be anything more objectionable, than the application of acid for the destruction, or extirpation of the nerve. He was decidedly of opinion, that extirpation was much preferable; as frequently alveolar abscess followed the use of acids. (*Hear, hear.*)

Mr. Bell observed, that the operation of the steel in destroying the nerve, was attended with very great pain, so great, that he was disposed to think very few would submit to it. He inserted the nitric acid on a small piece of soft wood; a sharp pain was the result, but one not so violent as that caused by the steel.

Mr. Perkins objected to wood, because it was clumsy, and did not go sufficiently far up. He would not venture to decide which mode should have the preference—touching the nerve with wood, or touching it with steel; but he was decidedly for steel, inasmuch as it went farther, and did its business in one second. As to the pain, it was intense while it lasted, whether caused by wood or steel. (*Hear, hear.*)

Mr. Bell wished to know, had Mr. Perkins tried wood?

Mr. Perkins had not.

Mr. Bell thought, under these circumstances, it was impossible Mr. Perkins could decide which was the better.

Mr. Perkins conceived, that one manifest advantage in steel was, that they could pass it up the entire cavity, while it was impossible to pass wood so far; or if they did, it would, in all probability, break. He had no doubt that Mr. Bell had succeeded in the use of wood; but he (Mr. Perkins) should not like to venture on it.

Mr. Bell had never known the wood to break.

Mr. Underwood thought, the application of the steel broach very useful; but the operation required a firm hand, and should be boldly and rapidly done. He had found it a very good plan to pass up into the cavity a fish-hook, with a straightened point, and with the barb to catch the nerve, when the operation was very instantaneous. He objected to the use of strong acids, because of the injury which they did to the dentine. (*Hear, hear.*)

Mr. David Hepburn had seen wooden plugs used with very uncertain effect in the taking of models, and that too in a vast number of cases. The difficulty was to get the wood cut so carefully as to mark a direct line to the orifice of the fang. He was in favor of having no pivot opening in the model at all, but of simply taking a model of the hole in wax; and if they got this, the immediate direction of the pin was not of so much consequence, as the slightest bending with the pliers would alter the direction so as to get the holes opposite. (*Hear, hear.*) Besides this, the character of the mouth was generally a quite sufficient indication of the direction of the fang. He should say, that he had listened with surprise to the remarkable statement made by an experienced member, as to his cutting off with an excising forceps a tooth in such a manner as to leave the stump fit for pivoting. He (Mr. Hepburn) had never seen a case in which it was not necessary to apply the file in some form or other. He should very much apprehend, that the use of such force as this operation with the excising forceps required, would break the fang, or bring away the root. As to having a gold tube to allow the pus to escape, such a tube must be very large, or very weak. He had seen many cases in which the decay was very considerable, and the wood plug was used, but he did not think wood a good material for the purpose in ordinary cases. As to the preparation of the material for the model, honey was a very good substitute for oil, and was not liable to the objection to the latter on account of its taste. With respect to the destruction of the nerve, he gave the preference to its destruction by steel. (*Hear, hear.*)

Mr. Mathews could assure Mr. Hepburn, that during the many years which he had been using the excising forceps, he had never known so untoward event as the breaking of the fang, or the coming away of the root of the tooth, to occur. But he had known this advantage to arise, that in cutting off the crown of the tooth, the nerve was carried off along with it. (*Hear, hear.*)

On the motion of Mr. Mathews, the further discussion of the subject of Mr. Perkins' paper was then adjourned till the following monthly meeting.

MONTHLY MEETING, *June* 2.

The discussion on Mr. Perkins' paper was resumed this evening.

Mr. Mathews exhibited his excising forceps, and also specimens of teeth cut off by that instrument.

Mr. Rymer observed that he had known several cases in which bicuspids had been pivoted ; but his opinion was, that as a general rule, it was not desirable to do so. (*Hear.*)

The President should say, that he had not been so fortunate in practice as his friend Mr. Mathews ; for while Mr. Mathews had never had an "untoward" case in cutting off a tooth for the purpose of pivoting, he (the President) was bound in candor to confess that he had had several. (*Hear.*)

Mr. Mathews had been misunderstood. When he said he had never had an "untoward" case, he meant that he had never been so unfortunate as to bring away the fang with the portion of the tooth intended to be excised. Some gentlemen had expressed their apprehensions that such a result would follow the use of his excising forceps ; and in answer to them, he said that he had never had so untoward a case. (*Hear.*)

Mr. Perkins thanked the members and associates for the interest they had manifested in the subject of his paper.

With many of the observations made by gentlemen who had taken part in the discussion he fully agreed; and in reply to what had been stated by others, he should say, that he still adhered to the views which he had ventured to put forward in his paper. (*Hear, hear.*)

A warm vote of thanks was then passed to Mr. Perkins, for his very able paper.

*The Electric Cautery, and its application to Dental Surgery.* Mr. T. Harding then read the following paper:

Mr. President and Gentlemen.—I cannot refrain, at the commencement of this paper, from observing, that it has been with great satisfaction I have come forward, as a member of the College of Dentists, to lay the result of my experience in dentistry before this body, and I trust, that the great bulk of our members will not fail to give the result of theirs in good time. The College of Dentists, I feel assured, is destined to become a great body in this country, and will not be behind its sister colleges in the United States of America, where dentistry occupies a distinguished rank, as well as in the province of Canada adjoining. Every good wish has been uttered for the prosperity and success of the College of Dentists of England, which many enterprising men have described as an institution which had long been wanted here, for the advancement of dental science. Our sphere of usefulness has already been extended by the delivery of lectures, which have been well attended; and a charter of incorporation is now only required to render the College of Dentists everything that its promoters and founders could wish.

It will be as well, at the commencement of the present communication, just to glance at the history of the introduction of the electric cautery, and its general application in surgical practice. Crusell, of St. Petersburg, was the first to employ it for surgical operations, although his researches generally, on its use, were not published before the year 1846, yet his operations bear date anterior to those of any other surgeon.

In 1844, M. Louyet, of Brussels, recommended the operation for destroying the dental nerve; and in 1845, Heider, of Vienna, at the instigation of Steinheil, of Munich, cauterized the dental nerves with the galvanic cautery. In 1850, Mr. Marshall was the first to employ it in practical surgery in this country, but his researches were not published before 1851, when he brought the subject before the Royal Medical and Chirurgical Society in April of that year. In the same year I published a short paper on its use in dental surgery, being the first to adopt it in that special branch of surgery in this country; and in the same journal, "The Lancet," a paper also appeared from Mr. Waite, recommending its use in dentistry.

Since 1851, it has been generally employed by others in England, France and Germany.

Being deeply impressed with the great value of this powerful agent, in many of the most delicate operations which come under the hand of the general surgeon, from reading the papers communicated to the Medico-Chirurgical Society by my friend, Mr. Marshall, giving in detail an account of the manner in which he employed the heat of electricity for the purpose of limited cauterization in surgical disease, an abstract of which appeared in "The Lancet," in May 1851; and, again, a subsequent report of several operations performed by him, in which the results showed most satisfactorily the great value of this new agent, it struck me that its introduction into the practice of dental surgery would prove of inestimable value, and of the greatest possible assistance, in effectually destroying the sensitive pulp of a decayed tooth, in a more certain, rapid, and safe manner than any of the numerous methods with which dentists are already familiar. I therefore conceived the idea, that a platinum wire, heated in the manner as recommended by Mr. Marshall, might be made available for the instantaneous destruction of an exposed tooth-pulp. I accordingly communicated my idea to Mr. Marshall, who fully concurred with me as to its importance. It had already suggested itself to his fertile mind, and had been mentioned by him as one of the obvious applications of his method of operating with the electric heat. His experience in this matter, moreover, enabled him to suggest for the purpose a very simple and suitable apparatus, which shall shortly be described.

In the first volume of "The Lancet," for 1851, there appeared a short communication from me on the destruction of the dental pulp by the heat of electricity, wherein I confidently stated that it might be regarded as a great advantage by all engaged in the practice of dental surgery. I had employed it for some months previous to the appearance of that paper, and fairly and justly claim to have been the first to use it in dental surgery in this country. In the same number of that journal was described an instrument for applying electric heat in dental operations, by Mr. Waite; but I had used it before the time mentioned by Mr. Waite, and moreover was not acquainted with his invention.

Now other methods have been employed for applying heat to destroy the nerve of an aching tooth. The old village doctress has long been famous for curing toothache by the thrust of a hot needle or pin into the tooth, and dentists have occasionally used a heated wire. The actual cautery has long been a practice in vogue, for the purpose of destroying the sensibility of the tooth pulp from caries, and has been generally performed by heating a long piece of steel, small at one end, but terminating in a bulbous head, about the size of a small pea, which is inserted into a handle. From the bulbous extremity projects a piece of platina wire, smaller or larger, according to circumstances; the bulbous end being heated in an ordinary lamp, until a red or white heat is obtained, communicates the heat to the platinum wire, which is then immediately used for the purpose required. Another method of applying hot wires to the teeth is by means of platinum sponge and hydrogen gas, known as œropile; but I have had no experience in the use of this.

In regard to the use of the actual cautery, let us see what Mr. Snell says of it in his book on the teeth: he says—"Even now, it (the destruction by the actual cautery) is frequently performed in an improper manner, which will account for the want of success which often attends it when attempted by ignorant men. As the operation is very generally performed, it would be more properly styled carbonizing the cavity of the tooth generally, than simply cauterizing the membrane." It must be obvious to every one, that the great cause of failure, such as is here described, depends upon the difficulty experienced in obtaining a sufficient and permanent amount of heat; for it is well known, that wire alone, which is the only substance sufficiently minute to be applied within the interior of the tooth, can retain the heat for but a very limited time. It is therefore necessary, in the hands of some dental surgeons, who are not satisfied with its effects, to apply it to the tooth, certainly more than once, perhaps several times in succession. This must prove of serious injury to the teeth, as it will carbonize a large surface of the tooth generally, instead of cauterizing, or destroying the tooth-pulp solely. Now all this is completely removed by the use of the electric cautery, which can never be surpassed for convenience and ready mode of application, besides possessing a steady, uniform, and constant degree of heat, which can be continued at pleasure until the proper effects are obtained, and then as magically discontinued by destroying the connection between the positive and negative wires. It has the advantage also, over every other known method of cauterizing, that it can be introduced into the patient's mouth, and actually place within the cavity of the tooth, before it is made to become incandescent—an advantage that cannot be over-estimated by those conscientious dental surgeons who are so frequently called upon to destroy a tooth-pulp.

*The nature of the apparatus* which I am in the habit of using may be thus described: I shall speak of the battery first, and the cauterizer after:—

The *battery* is a compound one of Smee's, and consists of six pairs of plates of zinc and platinized silver, contained in six cells, which are set in action by one fluid, viz. dilute sulphuric acid. The battery may of course vary according to the choice and taste of the operator, but it is desirable to render it as elegant and as simple in arrangement as possible. When I first employed the electric cautery, I used a battery of two pairs of plates in a single cell. I now prefer the larger battery of six cells, because a large battery with weak acid will last longer than a small one with strong acid; besides this, the action of the battery is more uniform, and lasts much longer. A Smee's battery is the most convenient of application; it is always clean, ready when wanted, and has the advantage moreover of cheapness. Grove's and Maynooth's batteries are not fitted for the purpose required, as they are troublesome, and often give out fumes of nitrous acid, which are decidedly objectionable.

The *cauterizer* is thus constituted: the terminal six inches of the poles, which are of copper wire plated, are supported on an ebony or ivory handle, upon the side of which one of the poles is interrupted at a particular point. The extremities of the poles are connected by a piece of platinum wire, a hundredth of an inch thick, and three-quarters of an inch long, which is bent into a loop. The sides of the loop are then brought parallel and nearly close to each other, without touching, and it is thus introduced into the pulp cavity of the tooth to be operated on. By a slight pressure on one side of the handle, the interrupted pole is temporarily joined, and the platinum wire immediately becomes brilliantly heated, as it lies in contact with the tooth pulp. Sometimes, however, I have found it desirable in the first place, to complete the galvanic current, and thus heat the platinum wire, before bringing it to bear upon the exposed pulp. The flexibility of the loop of wire enables the operator to bend it in any direction previously to use. In this way I have succeeded in rapidly destroying the pulps of decayed and condemned teeth, and have proceeded, sometimes after a few minutes, to the operation of filling with gold, or with other suitable stoppings, as Ash's metallic paste. I use several cauterizers with extremely thin wires, made expressly for myself by Coxeter, of Grafton street, and Maddox, of University street.

*Of the Operation in general.*—The affected tooth being carefully examined, its cavity is to be well dried out and cleaned; a soft napkin is then introduced, to protect the mouth from the possibility of contact with the instrument, the platinum point of which is passed into the cavity of the tooth; it is then heated, and, from its brilliancy, gives a clear and distinct light, and the tooth pulp is lightly touched with the heated wire, and the whole or particular portion of it required is destroyed. If the operator prefer it, he may have the wire heated before introduction into the mouth, but my own practice is generally to apply the wire before doing this, and then permitting incandescence to take place in the mouth, which gives a light which is not seen by the patient, and so well illuminates the interior of the tooth, as to permit the tooth pulp, or diseased membrane, to be seen very distinctly and clearly.

There is some caution to be observed in the use of this agent, which it will be as well to mention, and that is, to avoid burning or otherwise injuring the solid part of the tooth; particular attention and care should be paid to this point. This will not happen unless the application is prolonged, which will very rarely indeed be required, if special care be observed to have the wire at a white heat. This is the more necessary, to produce speedy destruction of the part to be touched, which is effected almost instantly. In one instance under my care, that of a lady for whom I nipped off the crown of an incisor tooth, for the purpose of fixing some artificial teeth, and so exposed the pulp of that tooth, I applied the electric cautery at barely a red heat, owing to feebleness of the acid; the consequence of this was, that the dental pulp became attached to the end of the wire, and was actually drawn out entirely. This has been preserved. It gave some slight pain for the moment, but nothing in comparison to the pointed steel or silver wire used by most dentists. This perhaps unimportant accident, I think, would not have occurred had the cautery been at a white heat, as it would then have completely carbonized or destroyed the part with which it came into contact.

The *effect* of the operation is the rapid destruction of the pulps of the decayed and condemned tooth; not the whole of the pulp, for that is not always necessary, but that portion of it especially which is exposed. If this is done with a light, steady hand, no subsequent inflammation is produced upon the substance of the tooth, or in the cavity. If there should be any marked sensitiveness in the tooth, independent of the pulp, the slightest application of the cautery to it will prove effectual in completely removing it. In the large number of cases in which I have employed the electric cautery, I have never known any bad effects produced on the tooth, and this I attribute to the care with which it has been applied. I am, however, quite prepared to believe that a want of attention in this respect would not only prove injurious to the tooth, but even in many instances cause its destruction. It would be only under such circumstances that the operation could be attended with or followed by severe pain.

In whatever condition the tooth-pulp may be, the operation is associated with a little pain. But as the time of its application is not unfrequently just a second or so, in the large majority of instances in which I have employed it, there has been no pain whatever felt. There may be a sort of a twinge, which is but momentary; and whatever pain may arise is not to be compared to that arising from the process of extracting a tooth, which as is well known, is by no means free from a very considerable amount of pain. Some of my patients have felt so little when it has been applied, that they have asked me to apply it a second time, to make all certain that the dental pulp has been effectually destroyed.

*The subsequent filling of the tooth* is a matter which demands attention after the pulp is destroyed. If the cavity is examined very minutely, a small black speck, or spot, can be seen after the cautery has been used; this is due to the carbonization of the pulp, and is a guide to some extent in the after process of removal of the carious portion of the tooth, which should always be done after the sensibility has been destroyed by the electric cautery, and is to be accomplished with care in the usual manner, taking the precaution to leave none of the tooth in that condition remaining; every particle of it should be removed. For a few days afterwards, sometimes only one, but generally two, the cavity is allowed to remain filled, with a combination of morphine and mastic, and then the tooth is stopped. Sometimes, again, I request my patients to allow a few days to elapse before I stop their teeth, the cavities being in the mean time filled with a solution of mastic and camphor. But I occasionally accomplish what I believe no other dentist has done, and that is, to plug, or stop the cavity in the same sitting during which the pulp has been destroyed. This, however, depends upon the complete absence of pain after the use of the cautery. I have already stated, that there is always a little pain, but sometimes this at once disappears, and I then do not hesitate to stop the teeth permanently. I also do this if there has been a little bleeding from the cavity previous to cauterization, stopping the tooth immediately. It might be supposed this procedure of stopping the teeth immediately after the destruction of the pulp, would be always followed by dull aching pain; but I am happy to say, that not the slightest indication of pain has, in the great majority of instances, ensued, in rather an extensive use of this powerful agent.

As a rule, however, the tooth should not be stopped on the same day as the electric cautery has been employed, unless in the exceptional instances just mentioned—the removal of the carious portion not being followed by sensibility. Experience and practice teach us to know the proper cases which can be plugged immediately.

By waiting a day or two, I have found, by experience also, that any sensibility remaining after the destruction of the dental pulp. and removal of the carious parts of the affected tooth, is sure to disappear, assisted by the solution of morphine and mastic, or mastic and camphor, which occupies the cavity. By this time the cavity will bear the pressure of an instrument within it, and an examination will show that the destroyed pulp has receded considerably inwards; this is apparent by noticing the black discoloration from the previous carbonization of the affected part, and as it is deeply situated, it is either out of the way of being pressed upon by the stopping of the tooth, or becomes a matter of the smallest possible importance, so far as my experience permits me to judge in this respect. I must, however, warn others not to mistake the black speck here referred to for actual caries.

Under the various circumstances which have been mentioned, the *results* of the operation are completely successful, and the teeth are serviceable for years. If, however, a tooth should remain tender after the use of the cautery, it is always better to wait for its complete disappearance before proceeding to stopping. I cannot call to mind any single instance in which the pain was at all persistent after its use, but it will be sure to become so, if the tooth is one not fairly suitable for preservation, from being either loose or diseased at the termination of a fang, such as a small fungous growth, or some similar cause. In such cases, as I will shortly show, the destruction of the tooth-pulp, accomplished no matter by what method, will prove unavailing and unsatisfactory, ultimate extraction in such instances proving the only resource. Should there be associated inflammation of the gums with a carious tooth, in which the pulp has been destroyed in the manner which has been recommended, then the usual means for combating it must be resorted to, such as a leech or two to the gum, and repeated fomentations with warm water alone, as I am in the habit of recommending, or with warm milk and water, or a poultice. For the pain in the tooth itself, morphine and mastic will be found quite sufficient.

Some patients express the receipt of immediate relief after the use of the cautery, others, again, not for an hour or two, but eventually they are quite relieved ; the pain, however, is, I repeat, extremely slight.

In the large number of instances in which I have used the electric cautery to destroy the sensitive tooth-pulp, I have not known an *accident*, in the true sense of the word, to happen, unless I should except the case in which the dental pulp was suddenly drawn out attached to the platinum wire, which I have preserved for illustration, and depending upon, as has been said, the wire being at a red instead of a white heat. Such a circumstance might occur again in the hands of others. To avoid injury or accidents to the teeth or gums, it is necessary to keep the hand quiet, firm and steady ; the heated wires, if suddenly dislodged, would assuredly burn the cheek or gums, the tongue or palate, especially if steadiness was not particularly observed on the part of the patient. It was, I may say, only the other day, that an accident of this kind happened to a lady, in whose tooth I was applying the cautery ; she suddenly turned her head, from some cause, when the heated wire touched the internal surface of the cheek. It may be observed, however, that so rapidly can the disconnection of the wires be accomplished, by removal of the finger from the handle of the instrument which is held in the hand, that an accident can really very seldom occur from the heated wire.

Having thus considered the history of the use of the electric cautery in general surgery, its application to dentistry, the nature of the apparatus employed, and the general features of the operaticn, together with its effects and results, I shall, in the next place, make a few observations upon the cases which are suitable, and upon those which are unfitted for its use ; and will then draw a comparison between the use of the cautery and other methods of destroying the pulp.

*Of the operation in particular.*—The great object of this operation, which I am endeavoring so strongly to recommend in the present communication, is to destroy the irritable ulcer in the membrane of the tooth which permits the nerve to be exposed, and which is often associated with the presence and even protrusion of minute granulations, not dissimilar to what the surgeon meets with as protruding from an ulcer situated over a carious or necrosed bone in some other part of the body. This condition is accompanied with a certain amount of inflammation and tumefaction. The cautery, as I have said before, completely destroys the affected parts, and there is no time left to produce a new surface; nor do I think that could be accomplished after the destruction of the pulp; nor, again, is it a necessary measure, as no particular good could be derived from it, were it to be permitted to occur. The cavity is at once plugged and the admission of air to the tooth prevented, and the sensitiveness and carious condition are permanently removed.

Now, of the *proper cases* in which the electric cautery may be used, the most important and common is that known as severe ordinary toothache, especially that form of tooth affection arising from a cavity with exposure of the tooth-pulp. In cases also of cavities requiring filling, in which too great a sensitiveness is present, thus preventing the satisfactory removal of the carious portions of the tooth, it is equally valuable. It is useful also in cases where the gums have receded, with exposure of a part of the neck of a tooth which is extremely sensitive and oftentimes very painful to the touch; in these it is necessary merely to touch the exposed necks, to remove the sensitiveness. It is not less serviceable in its application to tender, sensitive and bleeding gums, producing a new and healthy action, which permits them to become firmly attached again to the necks of the previously exposed teeth. Sometimes it is merely necessary to hold the heated wire near a sensitive neck, without actual contact, to remove the tenderness. I have destroyed, with the greatest ease and rapidity, the pulps of the incisor teeth which have been cut off for the purpose of being pivoted. Every dentist is aware of the sensitiveness which sometimes exists in a pivoted incisor tooth, depending upon the vitality of the stump; this is completely obviated by the use of the cautery. I have employed it also in numberless instances in which unusual sensitiveness exists to both warm and cold substances, depending upon a variety of causes—such as exposure of the necks ; or arising from chipping and fracture of the tooth from brittleness or some other cause ; or, again, where the tooth has been filed, cut, or accidentally broken. A very striking instance of this kind—that is, unusually great suffering from taking either warm or cold liquids into the mouth—came under my care but the other day, in which permanent and complete relief was afforded from the electric cautery. If a tooth is snapped off at the neck, and the pulp-cavity becomes exposed and painful, it can be destroyed, and an artificial tooth may be fastened to the stump, in the same manner as when the tooth is intentionally removed for this purpose. Sometimes, also, bleeding will occur from the rupture of some minute capillary vessels during the removal of caries from a cavity which may have become morbidly vascular, without the presence of actual pain or even sensitiveness ; it is equally serviceable here as in other forms of disease, and stops the hemorrhage, which is inconvenient and troublesome, from its interference with the progress of stopping the tooth. I have also applied it in sensitiveness, arising from the wearing away or grooving of a tooth from the constant pressure of the spring clasp of a plate, which has caused the destruction of the dentine ; mechanical friction, in fact, producing this condition.

As a general rule, the electric cautery may be used with decided advantage in almost every case of diseased tooth, with very few exceptions. But the commonest affection *constantly* requiring its use, I again repeat, is ordinary *toothache,* not unfrequently most agonizing in its character, and depending upon the presence of a cavity from caries, which has laid bare the delicate nervous pulp contained in it, which, so long as it is likely to come into contact with the liquids and solids of the mouth will continue in this condition. Destruction of the pulp and subsequent stopping prove the remedy ; the nerve is destroyed, the pain disappears, and the tooth remains for years, and answers as well as if it had been filled without exposure and destruction of the pulp.

The electric cautery, therefore, saves many a tooth, which without its aid, would be otherwise totally lost. It is preserved for years, and perhaps it is not saying too much, that if ordinary care and precaution are used, it will most probably last the lifetime of the individual. This, perhaps, may seem to be problematical, as many dentists would declare that if the nerve is destroyed, the tooth is dead ; it is a foreign body, and will last but a few years, ultimately decay, and require extraction. The cautery certainly destroys either a portion or the whole of the dental or nervous pulp. I will say, for argument sake, that it does completely and effectually destroy the nerve of the tooth. The tooth, however, receives nourishment from the periosteum covering its fang, as well as from that lining its socket, and it would seem that there may even be minute nervous twigs accompanying the equally minute capillary blood vessels which afford life and sustenance to the plugged tooth. It cannot, therefore, be looked upon as a foreign body, as it possesses and retains its vitality, which is derived through its fang, or in other words, from without, and is thus capable, from the reasons mentioned, of lasting with proper care the patient's lifetime. Mr. Nasmyth has beautifully shown that a tooth is supplied with a large number of minute nervous twigs and blood vessels.

I must also speak of another form of affection which has been relieved by it, namely, neuralgia of the face—a form of tic douloureux, supposed to depend upon some other cause than a carious tooth. On examining the mouth, however, I have detected an affected tooth, on destroying the pulp of which, with the cautery, and subsequently stopping it, a permanent cure has been effected.

It may be recommended as admirably suited to destroy fungous growths springing from the internal pulp, which often bleed very profusely on the slightest touch.

It will thus be seen that the application of the electric cautery is wide and extensive in the number and variety of cases of tooth disease.

The following however, *are unfit for it,* as it would not only produce no good, but harm might result from its use:

When a tooth is loose, with its external aspect sound, but the pain depending upon the presence of a fungous growth or small abscess developed at the end of one or more of the fangs. In such a case, extraction only will afford relief.

It will prove of no avail in an attack of inflammation of the central pulp, which may sometimes affect a tooth that is otherwise apparently sound. This may be known by the severe, heavy, throbbing pain which it occasions, running up to the head, accompanied with considerable tenderness of the tooth, and the gum around it. This condition may go on to suppuration of the pulp, or to abscess of the alveolus, and consequent death of the tooth. Leeches are here useful conjoined with some slight constitutional treatment.

And lastly, when we find a black, unsightly tooth lying loose in its socket, with pain depending upon the irritation produced by its presence, the cautery will be ineffectual, as the tooth is in a true state of necrosis, is quite dead, and has truly become a foreign body; it must, therefore, be removed. This condition I have not unfrequently seen to depend upon the use or abuse of mercury.

If a *comparison* be now instituted between the electric cautery, as I am in the habit of employing it for destroying the tooth pulp, and the numerous other means recommended to effect the same purpose, it will be seen that the balance of my confirmed judgment is entirely in favor of the former. Thus the great advantage of the cautery is that the desired effect is produced in less time than a minute; whilst it takes days, and even months, to accomplish destruction of the pulp by the various substances habitually in use: amongst these may be mentioned arsenic, used either alone, or in combination with other substances, as for instance, a mixture of equal parts of arsenic, morphine, and creosote; chloride of zinc in the solid form, or a combination of it and chloroform ; cobalt, chloroform, creosote, gun-cotton, tannin, and tannate of lead. Nitrate of silver is used, or nitric acid on a gold wire. Many other substances are employed. The nausea produced by the use of chloride of zinc, or nitrate of silver, is particularly disagreeable. I have, however, the strongest objection to the use of arsenic, more so than to any other substance ; and it is not without much thought on the subject, that I have come to the conclusion that it ought not to be employed in dental surgery. I have known instances in which this agent has been used, and the most acute and severe pain—in fact absolute torture—has followed for several days, before stopping the cavity could be accomplished. Besides the pain, there is also the danger arising from its absorption. A few months ago, a case of fatal poisoning appeared in the journals, which depended upon its absorption after employment in a dental operation. Added to the time required to destroy a tooth pulp by these various substances, there is also the mischief caused by their local application to the teeth themselves, which should not be lost sight of in the consideration of this question.

*Conclusions.*—I have now been in the habit of employing the electric cautery for upwards of six years, and during that time, have used it in more than five thousand instances, with an amount of success that has surpassed my most sanguine expectations, and without the occurrence of a single accident worthy of mention. I may truly say, that there are very few cases of toothache which cannot be relieved by it, when the membrane or tooth-pulp can be got at to destroy it. These very few cases are the exceptional instances which have already been referred to. It has been held by writers on diseases of the teeth, that the impossibility of instantaneously effecting the absolute destruction of the tooth-pulp in such teeth as are situated at the back of the mouth, which possess several diverging roots, is a sufficient ground for rejecting the means which were employed for that purpose—namely, the heating of a wire, in the form of the actual cautery, which cannot at any time be maintained at a white heat. It was in consequence of this very great difficulty that the actual cautery has fallen into disuse. Now, the advantage which the electric cautery has over every other conceivable method, is, that the white heat produced, and which cannot be obtained in any other manner, effects the purpose desired suddenly and with completeness. Besides this, it can be applied with perfect ease and freedom to teeth situated in any part of the mouth without the risk or danger of burning it—a risk almost impossible to avoid, with the greatest care, when the actual cautery or heated iron was employed.

It might be supposed, again, that some imaginary terror is likely to be excited in the patient's mind, at the idea of the electric cautery, in the shape of an incandescent body being used for any purpose in the mouth. I can truly say, that in the large amount of experience of its use which has fallen to my lot, that there is not the slighest ground for such a supposition. If this be an objection to its employment, then it falls to the ground, for I can call to mind no instance where this fear was manifested. Sometimes, as has been before mentioned, the wire is introduced already heated into the mouth, when being applied to the tooth.

At one time much dread of the actual cautery arose, from the burning of the mouth by the heated handle of the instrument, which was unavoidably employed to keep the wire hot, and this occasioned, we believe, its comparative rejection in this country. With the electric cautery such accidents are avoided; I may say, they scarcely ever occur, in the hands of any ordinary skillful man, for, owing to the extreme fineness of the wire employed, the local heat, though intense, is very limited in its action, and with due care the tooth substance need not suffer any appreciable injury. For the same reason, with the additional and more powerful one, of suddenly breaking the connection between the poles of the battery, no injury can happen to the mouth or gums.

Such, then, are the uses and advantages of this valuable agent in dental practice, and so well known is it becoming to a large number of the public, that a considerable proportion of my patients come recommended to me by medical men and others, for the express purpose of cauterization by it.

In March, a lady, to two of whose teeth I applied it instead of extracting them, writes to me, "Neither of my teeth have given me the least uneasiness. I am sure I have much to thank you for. Now, one need not fear the approach of toothache; we are so much more favored than those who lived a hundred years ago." These two teeth were, I may observe, cauterized and stopped at the same sitting.

I can now with still greater confidence, and much larger experience, lay this most valuable and efficient remedy before the notice of my professional brethren than I did in 1851. Very many years' constant trial have only further convinced me, that for ingenuity, simplicity of contrivance and application, nothing that has hitherto been invented can surpass it; and I again confidently leave it in their hands, gladly availing myself of my connection with the college of dentists, as one of its members, as a medium of doing so. (*Hear, hear.*)

I feel great pleasure in thanking you for your kind attention during, I fear, the tedious delivery of my paper on the electric cautery (*no, no,*) and at some future period I shall be most happy to prepare another paper, which I hope will meet with the same indulgence which you have so kindly extended to the one I have just read. (*Hear.*) Permit me again to express a hope, that every member of this college will not lose an opportunity of contributing his mental and physical energies for the benefit of this college, and the advancement of dental science, feeling as I do, that the prosperity of this excellent institution mainly depends on our united exertions. I mentioned, that I felt much indebted to Messrs. George Knight, of Foster Lane, for the apparatus and neatly constructed battery that they sent to the college of dentists. (*Hear, hear.*)—*Ib.*

## Figures and Tables

**Figure f1:**
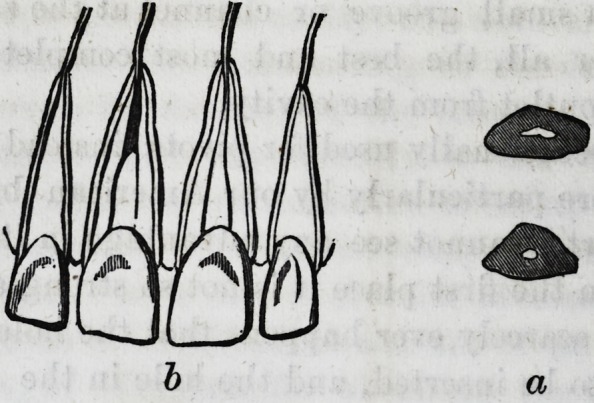


**Figures 2. f2:**
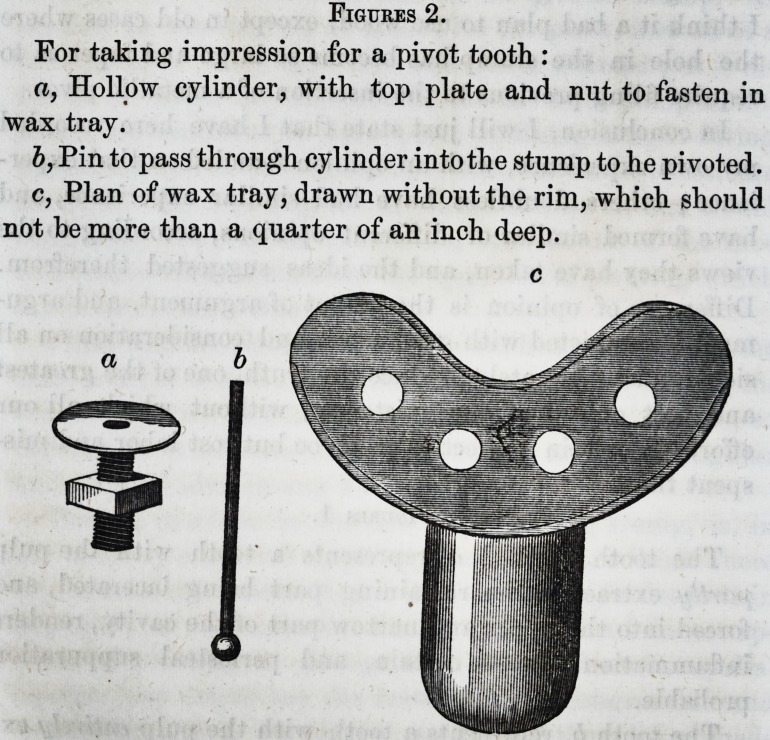


**Figures 3. f3:**